# ﻿*Acremoniumcapsici* and *A.guizhouense*, two new members of *Acremonium* (Hypocreales, Sordariomycetes) isolated from the rhizosphere soil of *Capsicumannuum*

**DOI:** 10.3897/mycokeys.95.97062

**Published:** 2023-01-04

**Authors:** Shuo-Qiu Tong, Lei Peng, Yong-Jun Wu

**Affiliations:** 1 College of Life Sciences, Institute of Agro-bioengineering, Guizhou University, Guiyang 550025, China Guizhou University Guiyang China; 2 Tea Academy, Guizhou University, Guiyang 550025, China Guizhou University Guiyang China

**Keywords:** filamentous fungi, morphology, new species, phylogeny, taxonomy

## Abstract

Two new species, *Acremoniumcapsici* and *A.guizhouense*, isolated from the rhizosphere soil of *Capsicumannuum*, are described and illustrated. Two-locus DNA sequences based on phylogeny, in combination with the morphology of the asexual morph, were used to characterize these species. In the phylogenetic tree, both new species clustered into a monophyletic clade with strong support, distinct from other previously known species of *Acremonium*. The new species differed from their allied species in their morphology.

## ﻿Introduction

*Capsicumannuum* L. is a globally grown and consumed spice crop that is rich in vitamins. *C.annuum* originated from the tropical and subtropical regions of Central and South America. It was introduced into China at the end of the Ming Dynasty, and has a long history of cultivation in China. According to the Food and Agriculture Organization of the United Nations, global *C.annuum* production reached approximately 36.1 million ton in 2020, with China producing the most in the world.

Link (1809) erected the genus *Acremonium*, whose members are geographically widespread and involve many substrates (Yang et al. 2019). As described by Gams (1971), the main diagnostic criteria of the genus *Acremonium* are conidiophores simple or verticillate; phialides narrow, solitary, generally cylindrical and gradually tapered towards the tips; conidia unicellular, hyaline to light-pigmented, spherical to cylindrical, arranged in slimy heads or unconnected chains or both; chlamydospores and sclerotia present or absent. The genus *Acremonium* is similar to some genera – *Sarocladium* W. Gams & D. Hawksw., *Brunneomyces* Giraldo, Gené & Guarro, and *Chordomyces* Bilanenko, M.L. Georgieva & Grum-Grzhimaylo etc. (Giraldo et al. 2015, 2017), including some of the simplest morphologies of all filamentous anamorphic fungi (Summerbell et al. 2011), so the morphological delimitation between them is challenging (Yang et al. 2019). Recent phylogenetic studies have documented that the genus *Acremonium* is polyphyletic, including sexual and nomenclaturally complex asexual morphs (Summerbell et al. 2011; Giraldo et al. 2012). To date, *Acremonium* has 219 records in the Index Fungorum (http://www.indexfungorum.org/Names/Names.asp, retrieval on Dec. 2022). However, many *Acremonium* taxa have been reported, but there are no trustworthy classification systems and little sequence data are available in GenBank for multigene analyses (Park et al. 2017). In the future, the classification of *Acremonium* will become clearer with the increase of molecular data.

In this study, seven strains of *Acremonium* were isolated in the process of investigating the rhizosphere fungal diversity of cultivated *Capsicumannuum* in Guizhou Province, southwest China, based on a culturable method. Identification of these strains in combination with morphological characteristics and phylogenetic analysis showed that these strains belong to two previously undescribed species of *Acremonium*. The new species differed from their allied species in their morphology.

## ﻿Materials and methods

### ﻿Fungal isolation and morphology

*Capsicumannuum* plants were cultivated in farmlands located in Guiyang, Guizhou Province, China (26°45'75"N, 106°64'87"E). One composite rhizosphere soil sample was taken from five randomly selected *C.annuum* plants. The roots were shaken vigorously to separate soil that is not tightly attached to the roots, and the remaining soil attached to the region 2–3 mm from the plant root was collected as the rhizosphere soil sample (Smalla et al. 2001). Fungi were isolated and purified using a dilution plate method as follows: 2 g samples were weighed with glass beads in a conical flask containing 20 mL sterile water, mixed evenly using eddy shock for 10 min, diluted to 1:10,000, and cultured on Martin’s medium supplemented with chloramphenicol and cycloheximide.

The purified isolates were transferred to potato dextrose agar (PDA), oatmeal agar (OA), malt extract agar (MEA), and corn meal agar (CMA) at 25 °C in darkness for 14 days to examine the macroscopic and morphological characteristics of the colonies. Photomicrographs of the diagnostic structures were obtained using an OLYMPUS BX53 microscope equipped with differential interference contrast optics, an OLYMPUS DP73 high-definition color camera, and cellSens software v.1.18. Both dry and living cultures were deposited at the Institute of Agro-bioengineering, Guizhou University.

### ﻿DNA extraction, PCR amplification, and sequencing

Total DNA was extracted from each of the new isolates using the BioTeke Fungus Genomic DNA Extraction kit (DP2032, BioTeke, Beijing, China) according to the manufacturer’s instructions. According to Li et al. (2022), the internal transcribed spacers (ITS), the 28S nrRNA locus (LSU), translation elongation factor 1-alpha gene region (TEF 1-α), RNA polymerase II second largest subunit gene (*RPB2*), and small subunit rDNA (SSU) were amplified and sequenced using ITS1/ITS4 (White et al. 1990), LROR/LR7 (Vilgalys and Hester 1990), EF1-983F/EF1-2218R (Rehner and Buckley 2005), fRPB2-5f/fRPB2-7cR (Liu et al. 1999), and NS1/NS4 (White et al. 1990) primers, respectively. All new sequences were submitted to GenBank (Table 1).

**Table 1.** Strains included in the present study.

Species	Strains	LSU	ITS	SSU	*TEF 1*-α	*RPB2*
*Acremoniumalternatum*	CBS 407.66 T	HQ231988	HE798150			
*Acremoniumalternatum*	CBS 831.97	HQ231989				
*Acremoniumarthrinii*	MFLU 18-1225 T	MN036334		MN036335	MN038169	
*Acremoniumbehniae*	CBS 146824 T	MW175400	MW175360			
*Acremoniumbiseptum*	CBS 750.69 T	HQ231998				
*Acremoniumblochii*	CBS 993.69	HQ232002	HE608636			
*Acremoniumborodinense*	CBS 101148 T	HQ232003	HE608635			
*Acremoniumbrachypenium*	CBS 866.73 T	HQ232004	AB540570			
*Acremoniumcamptosporum*	CBS 756.69 T	HQ232008		HQ232186		
*Acremoniumcavaraeanum*	CBS 101149 T	HF680202	HF680220			
*Acremoniumcavaraeanum*	CBS 111656	HF680203	HF680221			
*Acremoniumcavaraeanum*	CBS 758.69	HQ232012	HF680222			
*Acremoniumcerealis*	CBS 207.65	HQ232013				
*Acremoniumcerealis*	CBS 215.69	HQ232014				
*Acremoniumchiangraiense*	MFLUCC 14-0397 T	MN648329	MN648324			
*Acremoniumchrysogenum*	CBS 144.62 T	HQ232017		HQ232187		
*Acremoniumchrysogenum*	CBS 401.65	MH870276	MH858636			
*Acremoniumcitrinum*	CBS 384.96 T	HF680217	HF680236			
*Acremoniumcurvum*	CGMCC 3.20954 T	ON041050	ON041034	ON876754	ON494579	ON494583
*Acremoniumdimorphosporum*	CBS 139050 T	LN810506	LN810515			
*Acremoniumexiguum*	CBS 587.73 T	HQ232035				
*Acremoniumexuviarum*	UAMH 9995 T	HQ232036	AY882946			
*Acremoniumfelinum*	CBS 147.81 T	AB540488	AB540562			
*Acremoniumflavum*	CBS 596.70 T	HQ232037		HQ232191		
*Acremoniumflavum*	CBS 316.72	MH872204	MH860487			
*Acremoniumfuci*	CBS 112868 T		AY632653			
*Acremoniumfuci*	CBS 113889		AY632652			
*Acremoniumfusidioides*	CBS 109069	HF680204	HF680223			
*Acremoniumfusidioides*	CBS 991.69	HF680211	HF680230			
*Acremoniumfusidioides*	CBS 840.68 T	HQ232039	FN706542			
*Acremoniumglobosisporum*	CGMCC 3.20955 T	ON041051	ON041035	ON876755	ON494580	ON494584
*Acremoniumglobosisporum*	GZUIFR 22.037	ON041052	ON041036	ON876756	ON494581	ON494585
*Acremoniumglobosisporum*	GZUIFR 22.038	ON041053	ON041037	ON876757	ON494582	ON494586
*Acremoniumhansfordii*	CBS 390.73	HQ232043	AB540578			
*Acremoniumhennebertii*	CBS 768.69 T	HQ232044	HF680238			
*Acremoniuminflatum*	CBS 212.69 T	HQ232050				
*Acremoniummali*	ACCC 39305 T	MF993114	MF987658			
*Acremoniummoniliforme*	CBS 139051 T	LN810507	LN810516			
*Acremoniummoniliforme*	FMR 10363	LN810508	LN810517			
*Acremoniumparvum*	CBS 381.70A	HQ231986	HF680219			
*Acremoniumpersicinum*	CBS 310.59 T	HQ232077				
*Acremoniumpersicinum*	CBS 101694	HQ232085				
*Acremoniumpinkertoniae*	CBS 157.70 T	HQ232089		HQ232202		
*Acremoniumpolychroma*	CBS 181.27 T	HQ232091	AB540567			
*Acremoniumpotronii*	CBS 189.70	HQ232094				
*Acremoniumpseudozeylanicum*	CBS 560.73 T	HQ232101				
*Acremoniumpteridii*	CBS 782.69 T	HQ232102				
*Acremoniumpteridii*	CBS 784.69	HQ232103				
*Acremoniumsclerotigenum*	CBS 124.42 T	HQ232126	FN706552	HQ232209		
*Acremoniumsclerotigenum*	A101	KC987215	KC987139	KC987177	KC998961	
*Acremoniumsclerotigenum*	A130	KC987242	KC987166	KC987204	KC998988	
*Acremonium* sp.	E102	KC987248	KC987172	KC987210	KC998994	KC999030
*Acremoniumspinosum*	CBS 136.33 T	HQ232137	HE608637	HQ232210		
*Acremoniumstroudii*	CBS 138820 T		KM225291			
*Acremoniumtumulicola*	CBS 127532 T	AB540478	AB540552			
*Acremoniumvariecolor*	CBS 130360 T	HE608651	HE608647			
*Acremoniumvariecolor*	CBS 130361	HE608652	HE608648			
*Acremoniumverruculosum*	CBS 989.69 T	HQ232150				
***Acremoniumcapsici***	**SQT01 T**	**OP740978**	**OP703286**	**OP750190**	**OP757287**	**OP730522**
***Acremoniumcapsici***	**SQT02**	**OP740979**	**OP703287**	**OP750191**	**OP757288**	**OP730523**
***Acremoniumcapsici***	**SQT03**	**OP740980**	**OP703288**	**OP750192**	**OP757289**	**OP730524**
***Acremoniumguizhouense***	**SQT04 T**	**OP740981**	**OP703289**	**OP750193**	**OP757290**	**OP730525**
***Acremoniumguizhouense***	**SQT05**	**OP740982**	**OP703290**	**OP750194**	**OP757291**	**OP730526**
***Acremoniumguizhouense***	**SQT06**	**OP740983**	**OP703291**	**OP750195**	**OP757292**	**OP730527**
***Acremoniumguizhouense***	**SQT07**	**OP740984**	**OP703292**	**OP750196**	**OP757293**	**OP730528**
*Bryocentriabrongniartii*	M139	EU940105		EU940052		
*Bryocentriabrongniartii*	M190	EU940125		EU940052		
*Bryocentriametzgeriae*	M140	EU940106				
*Bulbitheciumhyalosporum*	CBS 318.91 T	AF096187	HE608634			
*Cephalosporiumpurpurascens*	CBS 149.62 T	HQ232071				
*Cosmosporalavitskiae*	CBS 530.68 T	HQ231997				
*Emericellopsisalkalina*	CBS 127350 T	KC987247	KC987171	KC987209	KC998993	KC999029
*Emericellopsisterricola*	CBS 120.40 T	U57082	U57676	U44112		
*Gliomastixroseogrisea*	CBS 134.56 T	HQ232121				
*Hapsidosporairregularis*	ATCC 22087 T	AF096192		AF096177		
*Kiflimoniumcurvulum*	CBS 430.66 T	HQ232026	HE608638	HQ232188		
*Lanatonectriaflavolanata*	CBS 230.31	HQ232157				
*Leucosphaerinaarxii*	CBS 737.84 T	HE608662	HE608640			
*Nigrosabulumglobosum*	ATCC 22102 T	AF096195				
*Paracremoniumcontagium*	CBS 110348 T	HQ232118	KM231831		KM231966	
*Parasarocladiumbreve*	CBS 150.62 T	HQ232005				
*Parasarocladiumradiatum*	CBS 142.62 T	HQ232104		HQ232205		
*Pestalotiopsishawaiiensis*	CBS 114491 T	KM116239	KM199339		KM199514	
*Pestalotiopsisspathulata*	CBS 356.86 T	KM116236	KM199338		KM199513	
*Pseudoacremoniumsacchari*	CBS 137990 T	KJ869201	KJ869144			
*Sarcopodiumvanillae*	CBS 100582	HQ232174	KM231780		KM231911	
*Sarocladiumbacillisporum*	CBS 425.67 T	HQ231992	HE608639	HQ232179		
*Sarocladiumbactrocephalum*	CBS 749.69 T	HQ231994	HG965006	HQ232180		
*Sarocladiumstrictum*	CBS 346.70 T	HQ232141	AY214439	HQ232211		
*Sarocladiumterricola*	CBS 243.59 T	HQ232046		HQ232196		
*Seliniapulchra*	AR 2812	GQ505992	HM484859		HM484841	
*Trichotheciumcrotocinigenum*	CBS 129.64 T	HQ232018	AJ621773			
*Trichotheciumindicum*	CBS 123.78T	AF096194		AF096179		
*Trichotheciumroseum*	DAOM 208997	U69891		U69892		
*Trichotheciumsympodiale*	ATCC 36477	U69889		U69890		

Notes: “T” stands for Ex-type strains. New isolates are in bold and blue.

### ﻿Phylogenetic analyses

In this study, we utilized sequence data mainly from recent publications (Yang et al. 2019; Li et al. 2022) and the sequenced new isolates (Table 1). According to Li et al. (2022) and Yang et al. (2019), *Pestalotiopsisspathulata* (CBS 356.86) and *P.hawaiiensis* (CBS 114491) were chosen as the outgroup taxa. The sequences were aligned using MAFFT v7.037 (Katoh and Standley 2013) and adjusted using MEGA 6.06 (Tamura et al. 2013). The aligned sequences of LSU and ITS were concatenated using PhyloSuite v1.16 (Zhang et al. 2020).

The best-fit substitution model was selected using the corrected Akaike information criterion, in ModelFinder (Kalyaanamoorthy et al. 2017). The maximum likelihood (ML) and Bayesian inference (BI) methods were used in the analysis. The ML analysis was implemented in IQ-TREE v1.6.11 (Nguyen et al. 2015) with 10,000 bootstrap tests, using the ultrafast algorithm (Minh et al. 2013). For the BI, MrBayes v3.2 (Ronquist et al. 2012) was used and Markov chain Monte Carlo simulations were run for 5,000,000 generations with a sampling frequency of every 500 generations and a burn-in of 25%. The above analyses were carried out in PhyloSuite v1.16 (Zhang et al. 2020).

## ﻿Results

### ﻿Phylogenetic analyses

**Figure 1.**
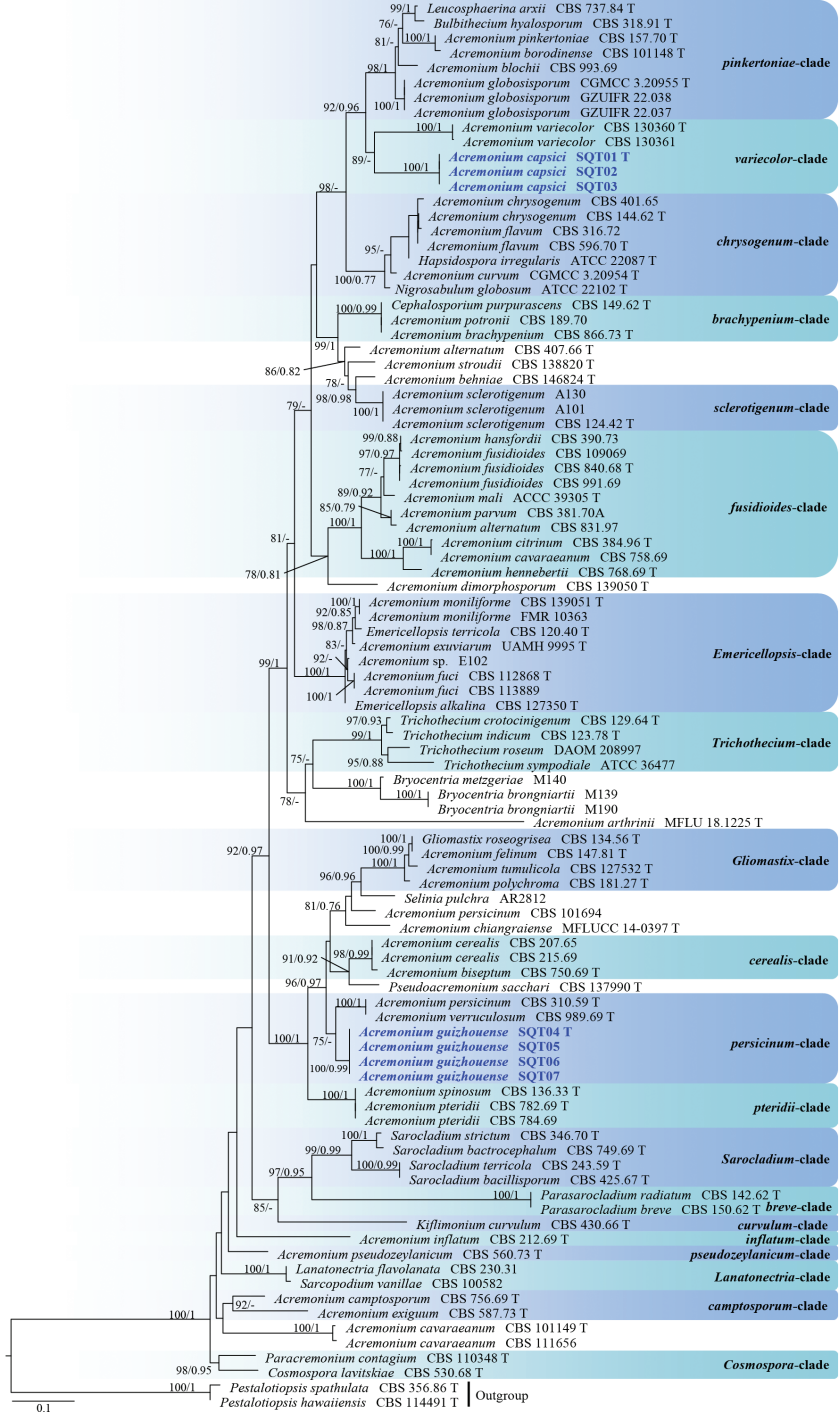
Phylogram generated from maximum likelihood analysis based on LSU + ITS sequence data. Bootstrap support values of maximum likelihood higher than 75% and Bayesian posterior probabilities greater than 0.75 are given above each branch. The new collection is highlighted in blue bold. Clades are identiﬁed using clade nomenclature formally deﬁned by Summerbell et al. (2011), and Yang et al. (2019). Ex-type strains are indicated by “T”.

Ninety-five isolates (including the seven with new sequence data) were included in our dataset (Table 1), which comprised 976 positions (including gaps), of which 377 were phylogenetically informative (122 of LSU and 255 of ITS). For Maximum-likelihood analyses, IQ-TREE’s ModelFinder under the corrected Akaike information criterion (AICc) proposed a TN+F+I+G4 for LSU, GTR+F+I+G4 for ITS. For Bayesian analysis, IQ-TREE’s ModelFinder under the AICc proposed a GTR+F+G4 for LSU, GTR+F+I+G4 for ITS. The results show that the isolates SQT01, SQT02, and SQT03 clustered in a single clade with high support (ML BS 100/BI pp 1), and were closely related to *Acremoniumvariecolor* (Fig. 1). The isolates SQT04, SQT05, SQT06, and SQT07 also clustered in a single clade with high support (100/0.98), and were closely related to *A.persicinum* and *A.verruculosum* (Fig. 1).

### ﻿Taxonomy

#### ﻿ Acremonium capsici

Taxon classificationFungiHypocrealesBionectriaceae

S.Q. Tong & Y.J. Wu sp. nov.

C5A6A4D7-BDBB-521D-B447-7A4B730B4FDA

846330

Fig. 2

##### Etymology.

Referring to the type strain isolated from the rhizosphere soil of *Capsicumannuum*.

##### Type.

Guiyang City, Guizhou Province, China 26°45'75"N, 106°64'87"E, isolated from the rhizosphere soil of *Capsicumannuum*, August 2022, Shuo-Qiu Tong (dried holotype culture SQT H-01, ex-holotype culture SQT01). GenBank: ITS = OP703286; LSU = OP740978; SSU = OP750190; *TEF 1*-α = OP757287; *RPB2* = OP730522.

##### Description.

Culture characteristics (14 days at 25 °C) – Colonies on PDA 20–21 mm diam, white, hairy, flat, radially striated, with a regular edge; reverse white. Colonies on MEA 18–19 mm in diameter, white, ﬂoccose, radially striated, with a regular edge; reverse white. Colonies on OA 18–19 mm in diameter, pale white, flat, with regular edge; reverse pale white. Colonies on CMA 18–19 mm in diameter, pale white, felty, with regular edge; reverse pale white. ***Hyphae*** hyaline, smooth, septate, branched, 1.0–2.5 µm wide. ***Phialides*** straight to flexuous, hyaline, smooth, arising from superﬁcial hyphae, from hyphal strands or from hyphal coils, 20–42 μm (n = 50) long, 1–2 μm (n = 50) wide at the base. ***Conidia*** arranged in slimy heads, one-celled, ovoid to ellipsoidal, fusiform, 2.0–3.5 × 1.5–2.0 µm (n = 50), hyaline, smooth, or rough. ***Chlamydospores*** and teleomorph were not observed.

##### Additional specimens examined.

Guiyang City, Guizhou Province, China 26°45'75"N, 106°64'87"E, isolated from the rhizosphere soil of *Capsicumannuum*, August 2022, Shuo-Qiu Tong, SQT02, *ibid.*, SQT03. GenBank: ITS = OP703287–OP703288; LSU = OP740979–OP740980; SSU = OP750191–OP750192; *TEF 1*-α = OP757288–OP757289; *RPB2* = OP730523–OP730524.

##### Known distribution.

Guiyang City, Guizhou Province, China.

##### Notes.

In a phylogenetic tree based on LSU + ITS sequences, *Acremoniumcapsici* forms a separate clade sister to *A.variecolor* in *Acremoniumsensu lato* (Bionectriaceae). In a comparison of LSU and ITS nucleotides, *A.capsici* (Type strain SQT01) has 93% and 83% similarity, in LSU (459/492 bp, one gap) and ITS (388/468 bp, 16 gaps), which is different from *A.variecolor* (CBS 130360). They are distinguished by the appearance of colonies on OA, MEA, and PDA: colonies of *A.capsici* grow slowly (less than 25 mm), and are white, while colonies of *A.variecolor* grow faster (more than 40 mm), and are white to yellowish (Giraldo et al. 2012). In addition, *A.capsici* bear simple phialides, while *conidiophores* of *A.variecolor* are mostly branched, bearing whorls of two to five phialides (Giraldo et al. 2012). *A.variecolor* produces sessile conidia, which is not seen in *A.capsici* (Giraldo et al. 2012).

**Figure 2.**
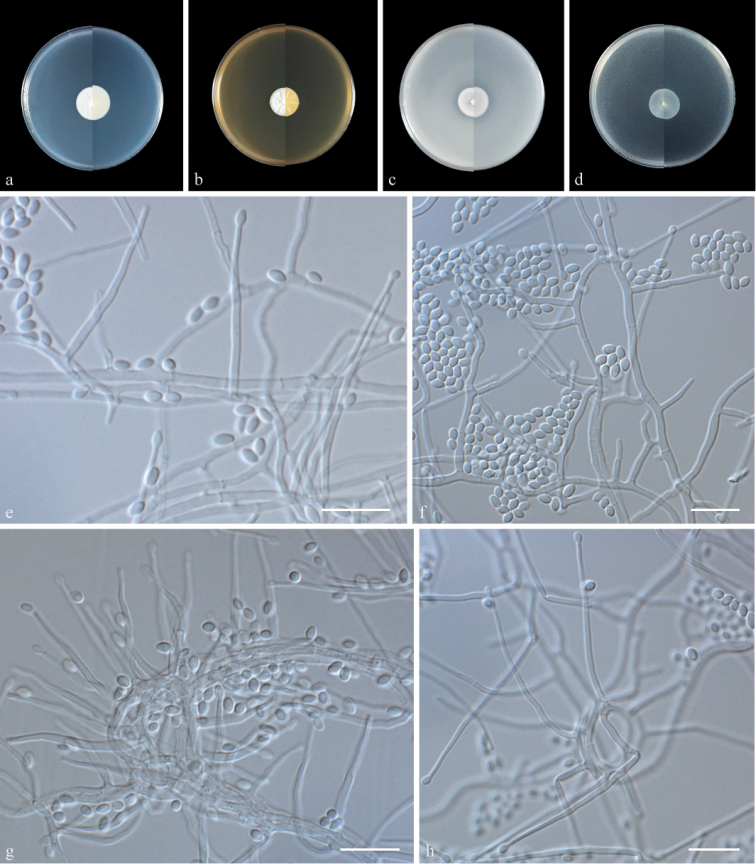
Morphology of *Acremoniumcapsici* sp. nov. **a–d** colony on PDA, MEA, OA, and CMA after 14 days at 25 °C (upper surface and lower surface) **e** phialides **f** conidia **g** phialides arising from ropes of hyphae **h** phialides arising from hyphal coils. Scale bars: 10 μm (**e–h**).

#### ﻿ Acremonium guizhouense

Taxon classificationFungiHypocrealesBionectriaceae

S.Q. Tong & Y.J. Wu sp. nov.

BD4EAA60-42B7-5B5F-8231-CC54D1DF1DA3

846331

Fig. 3

##### Etymology.

Referring to the country where this fungus was first isolated.

##### Type.

Guiyang City, Guizhou Province, China 26°45'75"N, 106°64'87"E, isolated from the rhizosphere soil of *Capsicumannuum*, August 2022, Shuo-Qiu Tong (dried holotype culture SQT H04, ex-holotype culture SQT04). GenBank: ITS = OP703289; LSU = OP740981; SSU = OP750193; *TEF 1*-α = OP757290; *RPB2* = OP730525.

##### Description.

Culture characteristics (14 days at 25 °C) – Colonies on PDA 16–19 mm in diameter, yellowish white to grayish yellow, flat, zonate, with regular edge; reverse brownish orange. Colonies on MEA 9–13 mm in diameter, yellowish white to white, compact, convex with papillate surface, margin dentate, aerial mycelia extremely sparse; reverse yellowish white to umber. Colonies on OA 14–16 mm in diameter, pale, felty, with regular edge; reverse pale white. Colonies on CMA 16–14 mm in diameter, pale white, felty, with regular edge; reverse pale white. ***Hyphae*** hyaline, smooth, septate, branched, 1.0–3.0 µm wide. ***Phialides*** straight to flexuous, hyaline, smooth, arising from hyphae, 15.5–33.5 μm (n = 50) long, 1.5–2.5 μm (n = 50) wide at the base. ***Conidia*** gathered in slimy heads, one-celled, ovoid to ellipsoidal, 2.5–3.0 × 3.5–5.0 µm (n = 50), hyaline, smooth or rough. ***Chlamydospores*** and teleomorph not observed.

##### Additional specimens examined.

Guiyang City, Guizhou Province, China 26°45'75"N, 106°64'87"E, isolated from the rhizosphere soil of *Capsicumannuum*, August 2022, Shuo-Qiu Tong, SQT05 = SQT06, *ibid.*, SQT07. GenBank: ITS = OP703290–OP703292; LSU = OP740982–OP740984; SSU = OP750194–OP750196; *TEF 1*-α = OP757291–OP757293; *RPB2* = OP730526–OP730528.

##### Known distribution.

Guiyang City, Guizhou Province, China.

##### Notes.

**Figure 3.**
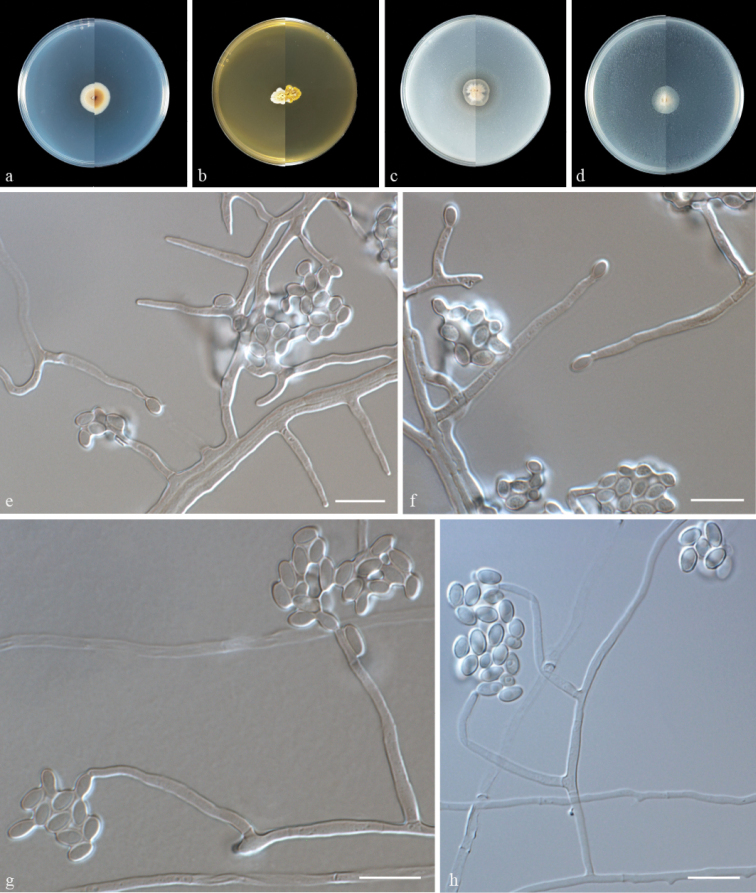
Morphology of *Acremoniumguizhouense* sp. nov. **a–d** colony on PDA, MEA, OA, and CMA after 14 days at 25 °C (upper surface and lower surface) **e, f** phialides and conidia **g, h** conidia are held together in slimy heads. Scale bars: 10 μm (**e–h**).

Phylogenetic and morphological data (Figs 1, 3) support our isolates SQT04–SQT07 as new species of *Acremonium*. *A.guizhouense* is phylogenetically closely related to *A.verruculosum* and *A.persicinum*. However, they can be distinguished by their sequence similarity (97% similarity, 10 base pairs (bp) differences and two gaps in 497 bp of LSU in *A.verruculosum* CBS 989.69; 98% similarity, 12 base pairs (bp) differences, and four gaps in 809 bp of LSU in *A.persicinum* CBS310.59). Since *A.verruculosum* and *A.persicinum* lack ITS sequences, it was not possible to compare *A.guizhouense* with them. Morphologically, the conidia of *A.verruculosum* are long ellipsoidal to cylindrical, rather than ovoid to ellipsoidal in *A.guizhouense* (Gams 1971). *A.verruculosum*, on the other hand, has larger conidia than *A.guizhouense* (5.6–6.0 × 2.3–2.5 µm vs. 2.5–3.0 × 3.5–5.0 µm) (Gams 1971). Furthermore, conidia of *A.verruculosum* are catenulate, fusiform, pyriform to ellipsoidal rather than arranged as slimy heads, ovoid to ellipsoidal in *A.guizhouense* (Gams 1971). The conidia of *A.guizhouense*, on the other hand, are smaller than that of *A.persicinum* (2.5–3.0 × 3.5–5.0 µm vs. 3.2–4.8 × 1.2–3.0 µm) (Gams 1971).

## ﻿Discussion

Traditionally, a polyphasic approach based on morphology, physiology, biochemistry, or reactions to chemical tests, has been used to differentiate species (Senanayake et al. 2020). Currently, many new fungal taxa have been reported based on DNA sequences. Phylogenetic analysis is becoming increasingly important in reporting new taxa of fungi, and has gradually become a mandatory component. However, many previously published fungal taxa lack DNA molecular data, and even specimens have been lost (Zhang et al. 2022). Thus, there are still many undetermined, questionable, or misidentified taxa that warrant taxonomic investigations (Summerbell et al. 2018). Since most species of the genus *Acremonium* have only LSU and ITS sequences Li et al. (2022), we used only ribosomal sequences (LSU + ITS) for phylogenetic analysis, while the sequencing of other loci was aimed at establishing a database for future studies.

Members of the genus *Acremonium* are geographically widespread and ecologically diverse, and seem to colonize all types of substrates, including endophytes, epiphytes, saprophytes, human and plant pathogens, lichens, insects, or arthropods taxa (Yang et al. 2019). In addition, *Acremonium* species have various functions, such as biological control (Shang et al. 2018), enhancing drought tolerance of grasses, and promoting nectar production of beans (Jaber and Vidal 2009), as well as improving plant resistance to plant pathogens (Kasselaki et al. 2006). In the present study, all the isolates were obtained from the rhizosphere soils of *Capsicumannuum*. Therefore, more studies are necessary to further confirm their relationship with their host plant *Capsicumannuum*.

In summary, seven isolates of *Acremonium* were obtained from the rhizosphere soils of *Capsicumannuum*. Morphological characteristics in combination with two-locus (LSU + ITS) phylogenetic analysis were used for delimitation. Therefore, two new species of *Acremoniumcapsici* (three isolates) and *Acremoniumguizhouense* (four isolates) are introduced. This study contributes to our understanding of the rhizosphere microbial population of *Capsicumannuum* and also of *Acremonium* species.

## Supplementary Material

XML Treatment for Acremonium capsici

XML Treatment for Acremonium guizhouense

## References

[B1] Gams W (1971) *Cephalosporium*-artige Schimmelpilze (Hyphomycetes). G. Fischer, Stuttgart.

[B2] Giraldo A Gené J Cano J de Hoog S Guarro J (2012) Two new species of *Acremonium* from Spanish soils. Mycologia 104(6): 1456–1465. 10.3852/11-402 22684288

[B3] Giraldo A Gené J Sutton DA Madrid H de Hoog GS Cano J Decock C Crous PW Guarro J (2015) Phylogeny of *Sarocladium* (Hypocreales). Persoonia 34(1): 10–24. 10.3767/003158515X685364 26240442 PMC4510268

[B4] Giraldo A Gené J Sutton DA Wiederhold N Guarro J (2017) New acremonium-like species in the Bionectriaceae and Plectosphaerellaceae. Mycological Progress 16(4): 349–368. 10.1007/s11557-017-1271-7

[B5] Jaber LR Vidal S (2009) Interactions between an endophytic fungus, aphids, and extrafloral nectaries: Do endophytes induce extrafloral-mediated defences in *Viciafaba*? Functional Ecology 23(4): 707–714. 10.1111/j.1365-2435.2009.01554.x

[B6] Kalyaanamoorthy S Minh BQ Wong TK von Haeseler A Jermiin LS (2017) ModelFinder: Fast model selection for accurate phylogenetic estimates. Nature Methods 14(6): 587–589. 10.1038/nmeth.4285 28481363 PMC5453245

[B7] Kasselaki AM Shaw MW Malathrakis NE Haralambous J (2006) Control of *Leveillulataurica* in tomato by *Acremoniumalternatum* is by induction of resistance, not hyperparasitism. European Journal of Plant Pathology 115(2): 263–267. 10.1007/s10658-006-9010-y

[B8] Katoh K Standley DM (2013) MAFFT multiple sequence alignment software version 7: Improvements in performance and usability. Molecular Biology and Evolution 30(4): 772–780. 10.1093/molbev/mst010 23329690 PMC3603318

[B9] Li X Zhang ZY Ren YL Chen WH Liang JD Pan JM Huang JZ Liang ZQ Han YF (2022) Morphological characteristics and phylogenetic evidence reveal two new species of *Acremonium* (Hypocreales, Sordariomycetes). MycoKeys 91: 85–96. 10.3897/mycokeys.91.86257 PMC9849060 36760887

[B10] Link HF (1809) Observationes in ordines plantarum naturales: Dissertatio I. Magazin. der Gesellschaft Naturforschenden Freunde Berlin 3: 3–42.

[B11] Liu YJ Whelen S Hall BD (1999) Phylogenetic relationships among Ascomycetes: Evidence from an RNA polymerse II subunit. Molecular Biology and Evolution 16(12): 1799–1808. 10.1093/oxfordjournals.molbev.a026092 10605121

[B12] Minh Q Nguyen M von Haeseler AA (2013) Ultrafast approximation for phylogenetic bootstrap. Molecular Biology and Evolution 30(5): 1188–1195. 10.1093/molbev/mst024 23418397 PMC3670741

[B13] Nguyen LT Schmidt HA von Haeseler A Minh BQ (2015) IQ-TREE: A fast and effective stochastic algorithm for estimating maximum-likelihood phylogenies. Molecular Biology and Evolution 32(1): 268–274. 10.1093/molbev/msu300 25371430 PMC4271533

[B14] Park SW Nguyen TTT Lee HB (2017) Characterization of two species of *Acremonium* (unrecorded in Korea) from soil samples: *A.variecolor* and *A.persicinum*. Mycobiology 45(4): 353–361. 10.5941/MYCO.2017.45.4.353 29371803 PMC5780367

[B15] Rehner SA Buckley E (2005) A *Beauveria* phylogeny inferred from nuclear ITS and EF1-α sequences: Evidence for cryptic diversification and links to *Cordyceps* teleomorphs. Mycologia 97(1): 84–98. 10.3852/mycologia.97.1.84 16389960

[B16] Ronquist F Teslenko M van der Mark P Ayres DL Darling A Höhna S Larget B Liu L Suchard MA Huelsenbeck JP (2012) MrBayes 3.2: Efficient Bayesian phylogenetic inference and model choice across a large model space. Systematic Biology 61(3): 539–542. 10.1093/sysbio/sys029 22357727 PMC3329765

[B17] Senanayake IC Rathnayaka AR Marasinghe DS Calabon MS Gentekaki E Lee HB Hurdeal VG Pem D Dissanayake LS Wijesinghe SN Bundhun D Nguyen TT Goonasekara ID Abeywickrama PD Bhunjun CS Jayawardena RS Wanasinghe DN Jeewon R Bhat DJ Xiang MM (2020) Morphological approaches in studying fungi: Collection, examination, isolation, sporulation and preservation. Mycosphere: Journal of Fungal Biology 11(1): 2678–2754. 10.5943/mycosphere/11/1/20

[B18] Shang SQ Chen YN Bai YL (2018) The pathogenicity of entomopathogenic fungus *Acremoniumhansfordii* to two-spotted spider mite, *Tetranychusurticae* and predatory mite *Neoseiulusbarkeri*. Systematic and Applied Acarology 23(11): 2173–2183. 10.11158/saa.23.11.10

[B19] Smalla K Wieland G Buchner A Zock A Parzy J Kaiser S Roskot N Heuer H Berg G (2001) Bulk and rhizosphere soil bacterial communities studied by denaturing gradient gel electrophoresis: Plant-dependent enrichment and seasonal shifts revealed. Applied and Environmental Microbiology 67(10): 4742–4751. 10.1128/AEM.67.10.4742-4751.2001 11571180 PMC93227

[B20] Summerbell RC Gueidan C Schroers H-J de Hoog GS Starink M Rosete YA Guarro J Scott JA (2011) *Acremonium* phylogenetic overview and revision of *Gliomastix*, *Sarocladium*, and *Trichothecium*. Studies in Mycology 68: 139–162. 10.3114/sim.2011.68.06 21523192 PMC3065988

[B21] Summerbell RC Gueidan C Guarro J Eskalen A Crous PW Gupta AK Gené J Cano-Lira JF van Iperen A Starink M Scott JA (2018) The protean *Acremonium*. *A. sclerotigenum/egyptiacum*: Revision, food contaminant, and human disease. Microorganisms 6(3): 88. 10.3390/microorganisms6030088 30115839 PMC6164869

[B22] Tamura K Stecher G Peterson D Filipski A Kumar S (2013) MEGA6: Molecular evolutionary genetics analysis version 6.0. Molecular Biology and Evolution 30(12): 2725–2729. 10.1093/molbev/mst197 24132122 PMC3840312

[B23] Vilgalys R Hester M (1990) Rapid genetic identification and mapping of enzymatically amplified ribosomal DNA from several Cryptococcus species. Journal of Bacteriology 172(8): 4238–4246. 10.1128/jb.172.8.4238-4246.1990 2376561 PMC213247

[B24] White TJ Bruns T Lee S Taylor J (1990) Amplification and direct sequencing of fungal ribosomal RNA genes for phylogenetics. In: Innis MA, Gelfand DH, Sninsky JJ, White TJ (Eds) PCR protocols: a guide to methods and applications, Academic Press, San Diego, California, 315–322. 10.1016/B978-0-12-372180-8.50042-1

[B25] Yang CL Xu XL Jeewon R Boonmee S Liu YG Hyde KD (2019) *Acremoniumarthrinii* sp. nov., a mycopathogenic fungus on *Arthriniumyunnanum*. Phytotaxa 420: 283–299. 10.11646/phytotaxa.420.4.4

[B26] Zhang D Gao FL Jakovlić I Zou H Zhang J Li WX Wang GT (2020) PhyloSuite: An integrated and scalable desktop platform for streamlined molecular sequence data management and evolutionary phylogenetics studies. Molecular Ecology Resources 20(1): 348–355. 10.1111/1755-0998.13096 31599058

[B27] Zhang ZY Ren YL Chen WH Liang JD Han YF Liang ZQ (2022) New taxonomic framework for Arthrodermataceae: A comprehensive analysis based on their phylogenetic reconstruction, divergence time estimation, phylogenetic split network, and phylogeography. Antonie van Leeuwenhoek 115(11): 1319–1333. 10.1007/s10482-022-01774-0 36018401

